# 1,2-Bis[(3-methoxyphenyl)methyl]ethane-1,2-Dicarboxylic Acid Reduces UVB-Induced Photodamage In Vitro and In Vivo

**DOI:** 10.3390/antiox8100452

**Published:** 2019-10-05

**Authors:** Po-Yuan Wu, Tzu-Yu Lin, Chien-Wei Hou, Qiao-Xin Chang, Kuo-Ching Wen, Chien-Yih Lin, Hsiu-Mei Chiang

**Affiliations:** 1 Department of Dermatology, China Medical University Hospital, Taichung 40402, Taiwan; wu.poyuan@gmail.com; 2 School of Medicine, China Medical University, Taichung 40402, Taiwan; 3 Department of Cosmeceutics, China Medical University, Taichung 40402, Taiwan; fishlin522@gmail.com (T.-Y.L.); nancystar597@gmail.com (Q.-X.C.); kcwen0520@mail.cmu.edu.tw (K.-C.W.); 4 Department of Biotechnology and Pharmaceutical Technology, Yuanpei University of Medical Technology, Hsinchu 30015, Taiwan; rolis.hou@mail.ypu.edu.tw; 5 Department of Medical Research, China Medical University Hospital, China Medical University, Taichung 40402, Taiwan; 6 Department of Biotechnology, Asia University, Taichung 41354, Taiwan; yihlin@asia.edu.tw

**Keywords:** 1,2-bis[(3-methoxyphenyl)methyl]ethane-1,2-dicaroxylic acid, photodamage, inflammation, nuclear factor-kappa B, inhibitor κB

## Abstract

This study investigated the effects and mechanisms of 1,2-bis[(3-methoxyphenyl)methyl]ethane-1,2-dicarboxylic acid (S4), a sesamin derivative, on anti-inflammation and antiphotoaging in vitro and in vivo. Human skin fibroblasts were treated with S4 and did not show cytotoxicity under concentrations of 5–50 µM. In addition, S4 also reduced ultraviolet (UV)B-induced intracellular reactive oxygen species (ROS) production. Additionally, S4 inhibited UVB-induced phosphorylation of mitogen-activated protein (MAP) kinases, activator protein-1 (AP-1), and matrix metalloproteinases (MMPs) overexpression. Furthermore, S4 also inhibited UVB-induced Smad7 protein expression and elevated total collagen content in human dermal fibroblasts. For anti-inflammatory activity, S4 inhibited UVB-induced nitric oxide synthase (i-NOS) and cyclooxygenase (COX)-2 protein expression and inhibited nuclear factor-kappaB (NF-ĸB) translocation into the nucleus. S4 ameliorated UVB-induced erythema and wrinkle formation in hairless mice. On histological observation, S4 also ameliorated UVB-induced epidermal hyperplasia and collagen degradation. S4 reduced UVB-induced MMP-1, interleukin (IL)-6, and NF-ĸB expression in the mouse skin. The results indicated that S4 had antiphotoaging and anti-inflammatory activities, protecting skin from premature aging.

## 1. Introduction

Aging is a progressive process leading to functional and esthetic changes in the skin. Skin aging is divided into intrinsic and extrinsic aging. Periodic and continuous exposure to ultraviolet (UV) radiation induces skin aging characterized by coarse wrinkles, hyperpigmentation, and sagging, which is known as photoaging [1,2]. Long-term exposure to UV can cause degradation of the extracellular matrix (ECM), including collagen and elastin in the dermis, and the presence of damaged collagen further down-regulates collagen synthesis [3,4]. Furthermore, fibroblasts may attach onto degraded collagen, causing inhibition of collagen synthesis. 

UV radiation exposure induces reactive oxygen species (ROS) generation, which induces damage to DNA, proteins, and lipids and produces inflammatory cytokines that activate several signal transduction pathways, causing skin damage and aging [5]. UV exposure triggers transcription factor activator protein-1 (AP-1) to activate matrix metalloproteinases (MMPs), resulting in the degradation of collagen and other connective tissue components [6]. MMPs are secreted in an inactive form, and the signal peptide and propeptide leave the catalytic domain of pro-MMP after activation, subsequently degrading ECM [7]. MMPs belong to a large family of proteases, and the major enzyme responsible for collagen digestion is MMP-1, also called interstitial collagenase. However, the activity of MMPs can be specifically inhibited by tissue inhibitors of metalloproteinases (TIMPs) [8]. The balance between TIMP and MMPs plays an important role in proteolytic remodeling of ECM in various physiologic situations, including matrix remodeling in the dermis [8].

The production of arachidonic acid increases in the skin after UV exposure, which results in skin inflammation, sunburn, and edema [9]. In addition, the generation of ROS attacks cell membranes, which produces prostaglandin E_2_ (PGE_2_) and nitric oxide (NO), causing skin erythema and inflammation [10]. UV exposure triggers another transcription factor, nuclear factor-kappaB (NF-κB) [11]. In general, NF-κB is inactive and bound with inhibitor κB (IκB) in the cytoplasm, whereas UV radiation or oxidative stress promote IκB ubiquitination and NF-κB will translocate into the nucleus and trigger inflammatory response of the skin [12]. Various cytokines are secreted, which upregulates inducible nitric oxide synthase (i-NOS) and cyclooxygenase (COX)-2 levels, which in turn consequently stimulates inflammation and skin damage [13]. 

**Figure 1:**
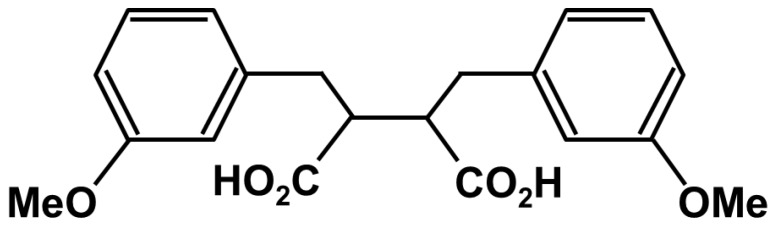
The structure of 1,2-bis[(3-methoxyphenyl)methyl] ethane-1,2-dicaroxylic acid (S4).

Sesame (*Sesamum indicum* L.) seed and oil is rich in various lignans, including sesamol, sesamin, sesamolin, and lignan glycosides, and has been reported to promote wound healing and to possess anti-inflammatory, antioxidative, and hypolipemic activities [14,15,16,17,18]. Sesamin has been found to exhibit antioxidative, anti-inflammatory, and antinociceptive activities [19,20,21]. Previous research also showed that sesamin scavenged ROS and NO and inhibited proinflammatory cytokine production, protecting the liver from injury [22]. One study found that a sesamin derivative, 1,2-bis[(3-methoxyphenyl)methyl]ethane-1,2-dicaroxylic acid (S4, Figure 1), had antioxidative activity and anti-inflammatory effects that contributed to neuroprotection [23]. The same study reported that S4 also neutralized ROS generation and reduced lipid peroxidation in BV-2 cells [23]. This current study attempted to investigate the activity and mechanism of S4 on photoprotection activity in skin cells and in animal models.

## 2. Materials and Methods

### 2.1. Chemicals and Materials

S4 was synthesized as previously described [23]. 3-[4,5-dimethylthiazole-2-yl]-2,5-diphenyltetrazolium bromide (MTT) was purchased from the USB Corporation (Cleveland, OH, USA). L-ascorbic acid, dimethyl sulfoxide, 2′,7′-dichlorofluorescin diacetate (DCFDA), leupeptin, phenylmethylsulfonyl fluoride, and paraformaldehyde were obtained from the Sigma-Aldrich Corporation (St. Louis, MO, USA). Dulbecco’s modified Eagle’s medium (DMEM), fetal bovine serum (FBS), penicillin, and streptomycin were purchased from Gibco, Invitrogen (Carlsbad, CA, USA). Enhanced chemiluminescence (ECL)™ Western blotting detection reagent was obtained from Amersham Biosciences (England). ProLong^®^ Gold antifade reagent with 4′,6-diamidino-2-phenylindole (DAPI) was purchased from Invitrogen (Carlsbad, CA, USA). Mitogen-activated protein (MAP) kinase inhibitors, including c-Jun N-terminal kinases (JNK) inhibitor II, PD98059, and SB203580, were obtained from Calbiochem (Darmstadt, Germany). Tris, sodium dodecyl sulfate (SDS), and Tween 20 were purchased from the USB Corporation (Cleveland, OH, USA). Other reagents and chemicals used in this study were reagent grade.

### 2.2. Cell Culture 

Human skin fibroblasts (Hs68) were purchased from the Bioresource Collection and Research Center (Hsinchu, Taiwan). Cells were seeded in DMEM containing 10% FBS and 100 U/mL penicillin/streptomycin and maintained in a humidity incubator at 37 °C with 5% CO_2_.

### 2.3. UVB Exposure

Cells covered with phosphate buffered saline (PBS) were exposed to 40 mJ/cm^2^ UVB from UV lamps (peak wavelength at 302 nm) in a CL-1000M UV crosslinker (UVP, Upland, CA, USA). The UVB exposure was performed as described in previous research [24].

### 2.4. Cell Viability Assay

Cell viability was determined with an MTT assay, and Hs68 were seeded in 24-well plates and subjected to various treatments. Various concentrations of S4 were added to the wells for 24 h after UVB exposure. The culture medium was replaced with 300 μL of 500 μg/mL MTT solution. After incubation for 3 h, the produced formazan was solubilized in 200 μL of 10% SDS in water. The absorbance was measured at 570 nm using a spectrophotometer (Tecan, Grodig, Austria). 

### 2.5. Intracellular ROS Concentration

Intracellular ROS generation was determined by a DCFH2DA fluorescence assay as described previously [24]. The cells were subjected to UV radiation after seeding for overnight and treated with various concentrations of S4. DCFH2DA was added to the cells and incubated for 30 min. Fluorescence was detected at an emission wavelength of 520 nm and excitation wavelength of 488 nm using a microplate reader (Thermo Electron Corporation, Vantaa, Finland). The results were expressed as the fold increase compared with the control. 

### 2.6. Western Blot Analysis

The protein levels of Hs68 cells after UVB irradiation and S4 treatment were assayed through Western blotting [24,25]. Hs68 fibroblasts were placed on a 10-cm dish for different treatments for 24 h. To isolate total protein, cultured Hs68 were washed with cold PBS and lysed using lysis buffer. The supernatant was collected using centrifugation at 12,000 *g* for 10 min. The protein concentrations were determined using the Bradford reagent (Bio-Rad Laboratories, Irvine, CA, USA). Samples containing equal amounts of protein (30 μg) were loaded and analyzed by Western blotting. The proteins were separated in 10% sodium dodecyl sulfate–polyacrylamide gel electrophoresis (SDS-PAGE) gels and transferred onto polyvinylidene difluoride (PVDF) membranes (Millipore, Bedford, MA, USA). PVDF membranes were blocked with blocking buffer (5% milk in Tris-buffered saline (TBST)) for at least 1 h at room temperature. Membranes were incubated with primary antibodies: anti-MMP-1 (Gene Tex, GTX100534), MMP-3 (Santa cruz, sc-21732), anti-MMP-9 (Abcam, ab38898), anti-TIMP-1 (Gene Tex, GTX112096), anti-c-Fos (Santa cruz, sc-7202), anti-c-Jun (Gene Tex, GTX61092), anti-p-c-Jun (Cell signaling, #9164), anti-extracellular signal–regulated kinases (ERK) (Santa cruz, sc-93), anti-*p*-ERK (Santa cruz, sc-16982), anti-JNK (Santa cruz, sc-46006), anti-*p*-JNK (Santa cruz, sc-12882), anti-p38 (Santa cruz, sc-7972), anti-*p*-p38 (Cell signaling, #4511), anti-Smad3 (Gene Tex, GTX61361), anti-Smad7 (Gene Tex, GTX 63201), anti-i-NOS (Abcam, ab129372), anti-COX-2 (Santa cruz, sc-19999), and anti-actin (Santa cruz, sc-1616) on an orbital shaker overnight. The membranes were incubated with horseradish peroxidase-linked secondary antibodies. An enhanced chemiluminescence Western blotting detection system (Fujifilm, LAS-4000, Tokyo, Japan) was used to determine the presence of the proteins, and densities were determined using a densitometric program (MultiGauge V2.2, Fuji Pharma, Tokyo, Japan).

### 2.7. Immunofluorescence Staining

Cells were seeded on coverslips overnight. S4 at various concentrations was added to the cells after UVB irradiation and then incubated for 24 h. Cells were fixed with 4% paraformaldehyde for 30 min, and incubated in 5% non-fat milk with 0.3% Triton X-100 of PBS buffer for 1 h. After being washed with PBS, the cells were incubated with antibody: anti-NF-κBp65 (Cell signaling, #8242), and incubated at 4 °C for 16 h. After another PBS wash, the secondary antibodies were added to the cells and incubated for 2 h. Finally, the cells on the coverslip were stained with ProLong Gold antifade reagent, examined under a fluorescence microscope, and fluorescence was detected at excitation wavelength of 488 nm (Nikon Eclipse 80i, Nikon Instruments Inc., Tokyo, Japan).

### 2.8. Determination of Total Collagen Contents

Total collagen synthesis in fibroblasts was detected using a Sircol^TM^ collagen detection kit as described previously [26,27]. After UVB exposure and S4 treatment, the cells were harvested and mixed with isolation and concentration reagents and incubated overnight. The Sircol^TM^ dye was added to the sample and incubated for 30 min. After centrifugation, ice-cold acid–salt washing reagent was mixed with the pellets, and the sample was centrifuged again. An alkali reagent was added to dissolve the precipitate, and the absorbance was detected at 555 nm.

### 2.9. Effect of S4 on UVB-Induced Photodamage in Hairless Mice

#### 2.9.1. Animals

Protocols for animal experiments were approved (104-238-B) by the Institutional Animal Use and Care Committee of China Medical University. Female BALB/cAnN.Cg-Foxn1nu/CrlNarl mice at 5 weeks old were purchased from the National Laboratory Animal Center in Taipei, Taiwan. Mice were kept in the animal center of China Medical University and accommodated for 1 week. 

#### 2.9.2. Experimental Design

Animals were randomly divided into the following five groups: non-UVB radiation and non-S4 treatment (normal), UVB-radiated, vehicle-treated and UVB-radiated (vehicle), UVB-radiated and 50-μM-S4-treated (UVB + 50 μM S4), and UVB-radiated and 200-μM-S4-treated (UVB + 200 μM S4) groups. Animals were exposed to gradient increased doses of UVB irradiation as described previously [28,29]. PEG400 (50 μL) was topically applied on the dorsal skin after UVB exposure in the vehicle-treated group daily, and 50 μL of 50 and 200 μM S4 dissolved in PEG400 was applied to the S4 groups. The a* values was detected every two weeks by a spectrophotometer (SCM-108, Laiko company, Tokyo, Japan). After 10 weeks of treatment, transepidermal water loss (TEWL) was measure by using an MPA 580 system (Courage+Khazaka Electronic GmbH, Cologne, Germany). The exposed areas of dorsal skin were excised and then immersed in 10% formaldehyde in PBS. The skin slides were mounted on a coverslip, stained in hematoxylin and eosin (H&E) or Masson’s trichrome, and examined under a microscope or used for immunohistological analysis as previously described [29].

#### 2.9.3. Immunohistological Analysis

The skin samples were incubated with primary antibodies for MMP-1, IL-6, NF-κB, and i-NOS. After being washed with PBS twice, the skin slides were incubated with the secondary antibody. The samples were examined under a microscope.

### 2.10. Statistical Analysis

Each analysis was carried out at least in triplicate in vitro study and the results were given as mean ± standard deviation [30]. Data between different groups were analyzed statistically using the Student’s *t* test or one-way ANOVA, followed by Tukey’s test. *p* < 0.05 was considered to be significant. All analyses were performed with GraphPad Prism 5 software (GraphPad Software Inc., La Jolla, CA, USA).

## 3. Results

### 3.1. S4 Did Not Cause Cytotoxicity in Hs68 Cells

**Figure 2:**
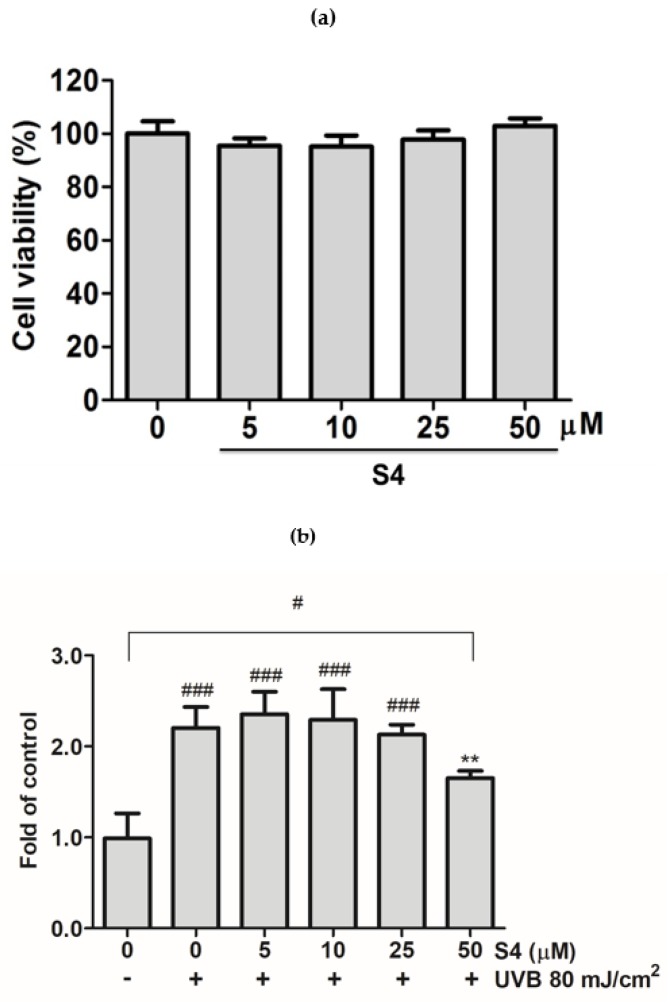
(**a**) Cell viability (%) of S4 in human skin fibroblasts. (**b**) Effect of S4 on intracellular oxidative stress in ultraviolet (UV)B-induced human skin fibroblasts. The results were given as mean ± standard deviation (*n* = 3). Significant difference versus non-irradiation group: ###, *p* < 0.001. Significant difference versus non-treatment group: **, *p* < 0.01.

To identify the effect of S4 on Hs68, the cytotoxic effect of S4 after 24 h of incubation was measured using an MTT assay. The MTT assay results showed that cell viability was over 95% after S4 treatment compared with the untreated control. The results indicated that S4 did not produce cytotoxic effects in Hs68 cells (Figure 2a). 

### 3.2. S4 Inhibited UVB-Induced Intracellular ROS Formation in Hs68 Cells

ROS production in skin fibroblasts was detected by DCFDA staining and examined under a plate reader. After UVB irradiation, intracellular ROS was substantially increased. However, ROS formation was considerably inhibited after treatment with S4 at 50 μM (Figure 2b). These results indicated that S4 could reduce UVB-induced intracellular ROS formation in Hs68 cells.

### 3.3. Effects and Mechanisms of S4 on UVB-Induced Skin Damage

#### 3.3.1. S4 Inhibited UVB-Induced Overexpression of MMPs

**Figure 3:**
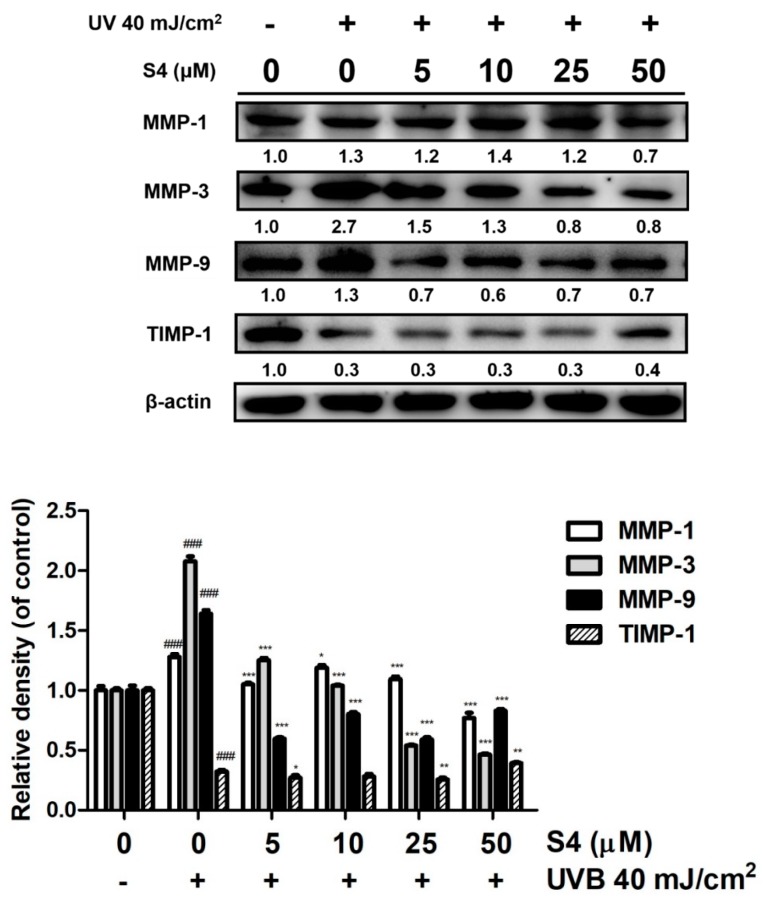
Effect of S4 on UVB-induced matrix metalloproteinases (MMPs) and tissue inhibitors of metalloproteinase (TIMP)-1 expression in human skin fibroblasts. The results were given as mean ± standard deviation (*n* = 3). Significant difference versus non-irradiation group: ###, *p* < 0.001. Significant difference versus non-treatment group: **, *p* < 0.01; **, *p* < 0.01; ***, *p* < 0.001.

UV radiation resulted in overexpression of MMP-1, -3, and -9 by 1.3-, 2.7-, and 1.3-fold compared with that of the untreated control; however, pretreatment with 5–50 μM S4 decreased MMP-1, -3, and -9 expression in Hs68 cells (Figure 3). S4 at doses over 50 μM significantly decreased the expression of MMP-1 by 0.7-fold compared with that of the untreated control, that at a dose of 25 μM significantly reduced MMP-3 expression by 0.8-fold, and that at a dose of 5 μM significantly reduced MMP-9 by 0.4-fold compared with that of the untreated control group (Figure 3). Based on these results, S4 could inhibit UV-induced MMP-1, -3, and -9 overexpression. 

#### 3.3.2. S4 Increased TIMP Expression

TIMP-1 is a natural inhibitor of MMPs and UVB exposure reduced TIMP-1 in the dermis (Figure 3). S4 treatment could increase the protein expression of TIMP-1 to protect skin from UVB-irradiation-induced skin damage. 

#### 3.3.3. S4 Inhibited UVB-Induced AP-1 Overexpression

**Figure 4:**
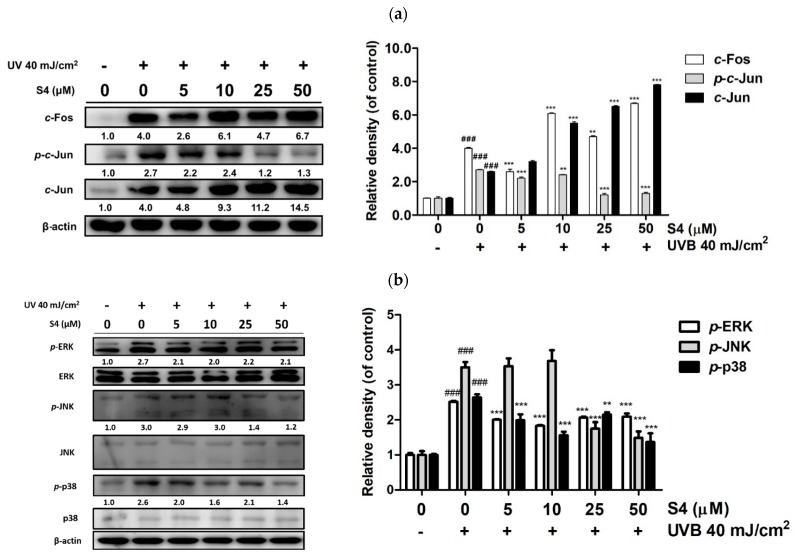
Effect of S4 on UVB-induced (**a**) AP-1 and (**b**) phosphorylation of mitogen-activated protein (MAP) kinases in human skin fibroblasts. The results were given as mean ± standard deviation (*n* = 3). Significant difference versus non-irradiation group: ###, *p* < 0.001. Significant difference versus non-treatment group: **, *p* < 0.01; ***, *p* < 0.001.

UVB exposure upregulated the expression of c-Fos by 4.6-fold compared with the untreated control, *p*-c-Jun by 2.7-fold, and that of c-Jun by 2.6-fold. However, treatment with S4 at 5 μM significantly decreased the expression of c-Fos by 2.6-fold, and 25 μM significantly decreased the expression of p-c-Jun by 1.2-fold (Figure 4a). 

#### 3.3.4. S4 Inhibited the UV-Induced Overexpression of MAP Kinases

UVB radiation induced MAP kinase activation, which resulted in the upregulation of MMPs. The protein expression levels of *p*-ERK, *p*-JNK, and *p*-p38 were 5.2-, 1.7-, and 3.0-fold after UVB radiation, respectively, compared with the untreated control (Figure 4b); nevertheless, this effect was substantially inhibited after treatment with S4. S4 at 50 μM considerably reduced *p*-ERK and *p*-p38 expression and *p*-JNK expression at doses over 10 μM.

#### 3.3.5. S4 Modulated the Expression of Smad3 and Smad7

**Figure 5:**
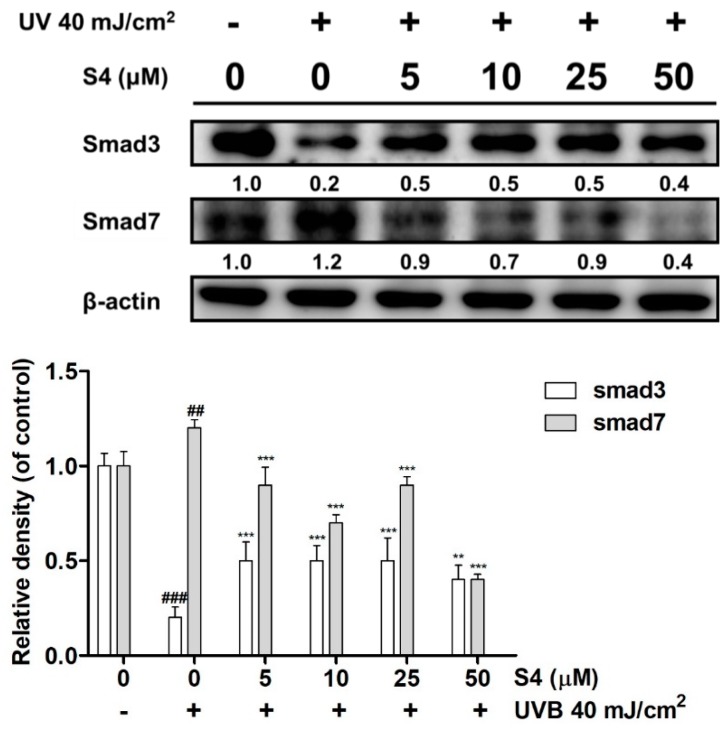
Effect of S4 on UVB-mediated Smad3 and Smad7 expression in human skin fibroblasts. The results were given as mean ± standard deviation (*n* = 3). Significant difference versus non-irradiation group: ##, *p* < 0.01; ###, *p* < 0.001. Significant difference versus non-treatment group: **, *p* < 0.01; ***, *p* < 0.001.

UVB radiation reduced Smad3 expression and increased Smad7 expression in Hs68 cells (Figure 5). UVB exposure decreased Smad3 protein expression by 0.2-fold compared with that of the untreated control, whereas S4 at 5 μM increased it by 0.5-fold compared with that of the untreated control. Smad7 expression was increased by 1.2-fold relative to the control group after UVB exposure and reduced by 0.9-fold compared with that of the control group after 5 μM S4 treatment (Figure 5).

#### 3.3.6. S4 Reversed UVB-Reduced Total Collagen Biosynthesis in Hs68

**Figure 6:**
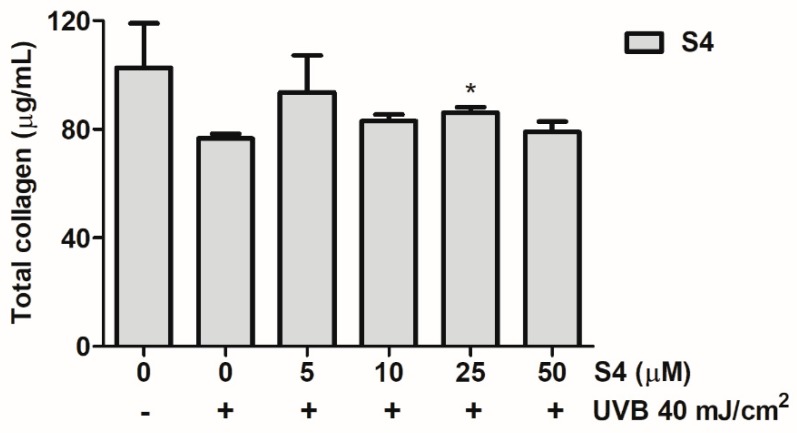
Effect of S4 on total collagen synthesis in human skin fibroblasts. The results were given as mean ± standard deviation (*n* = 3). Significant difference versus non-treatment group: *, *p* < 0.05.

Total collagen production was 102.7 ± 16.4 μg/mL in the untreated control and decreased to 76.7 ± 1.7 μg/mL with UVB radiation (Figure 6). However, S4 promoted collagen biosynthesis, and S4 treatment at 5 μM resulted in a collagen level of 93.7 ± 13.5 μg/mL (Figure 6). According to the results mentioned previously, S4 modulated Smad3/7 protein expression to increase collagen synthesis and protect skin from photodamage.

### 3.4. Anti-Inflammatory Effect of S4

#### 3.4.1. S4 Ameliorated UVB-Induced i-NOS and COX-2 Overexpression 

**Figure 7:**
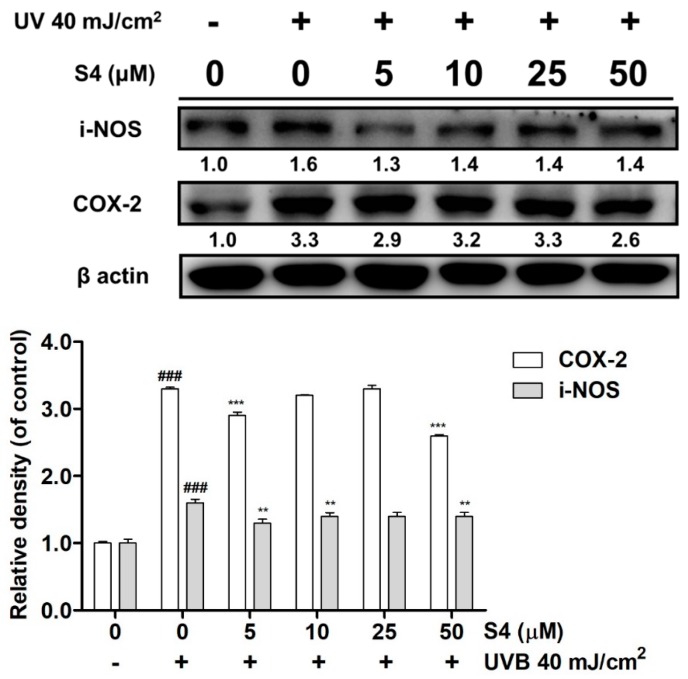
Effect of S4 on UVB-induced i-NOS and COX-2 in human skin fibroblasts. The results were given as mean ± standard deviation (*n* = 3). Significant difference versus non-irradiation group: ###, *p* < 0.001. Significant difference versus non-treatment group: **, *p* < 0.01; ***, *p* < 0.001.

After UVB radiation, the protein expressions of i-NOS and COX-2 were increased by 1.6- and 3.3-fold relative to those of the untreated control (Figure 7). Treatment with S4 at 5 μM, reduced the i-NOS protein expression levels by 1.3-fold of untreated control. Moreover, S4 at 50 μM reduced COX-2 expression in fibroblasts by 2.6-fold compared with that of the untreated control.

#### 3.4.2. S4 Inhibited UVB-Induced NF-κB Translocation into the Nucleus

**Figure 8:**
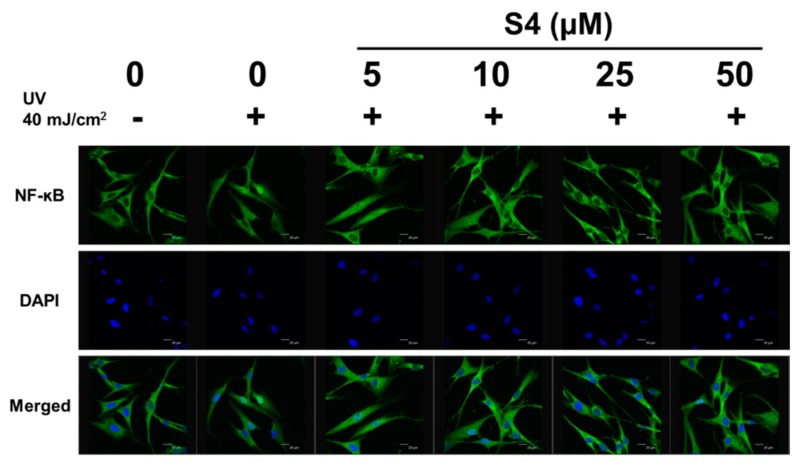
Effect of S4 on UVB-induced activation of nuclear factor kappa-B p65 in human skin fibroblasts.

UVB exposure promotes NF-κB activation and translocation into the nucleus of Hs68 cells, causing inflammation. After treatment with S4, the translocation of NF-κB was diminished (Figure 8). The results suggested that S4 can inhibit UVB-induced inflammation in skin cells.

### 3.5. S4 Ameliorated UVB-Induced Damage in Mouse Skin

#### 3.5.1. S4 Reduced UVB-Induced Skin Erythema

**Figure 9:**
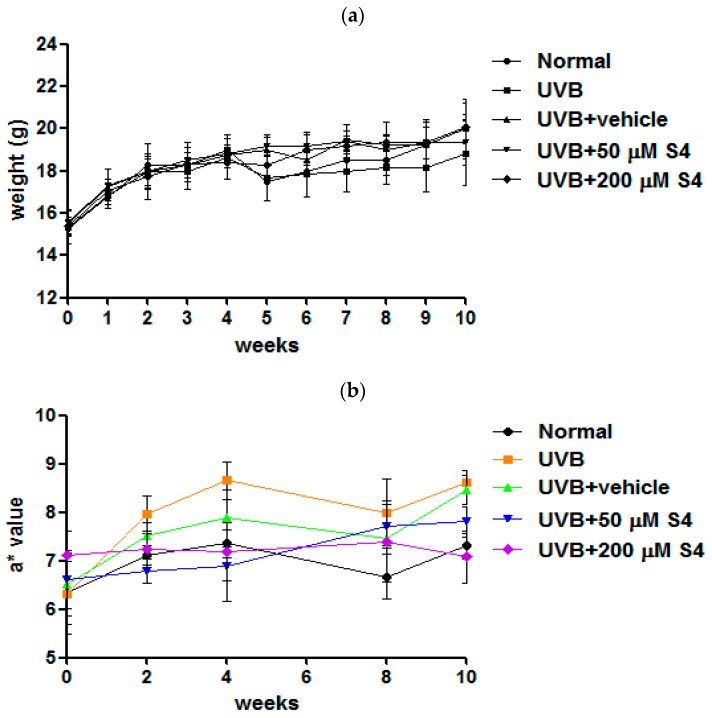
(**a**) Body weight of hairless mice over 10 weeks. (**b**) Effect of S4 on a* value in chronic UVB-irradiation hairless mice.

No significant differences in body weight were found between the groups after 10 weeks of UV exposure and S4 treatment (Figure 9a). The a* value indicated the degree of erythema and inflammation. In this study, the a* values increased at the second week of UVB exposure, indicating that UVB exposure caused erythema and inflammation (Figure 9b). The a* values observed for the UVB-irradiated and 200-μM-S4-treated mice at the 10th week were similar to those observed for the normal mice, indicating that S4 treatment decreased skin erythema and inflammation.

#### 3.5.2. S4 Reduced UVB-Induced TEWL 

**Figure 10:**
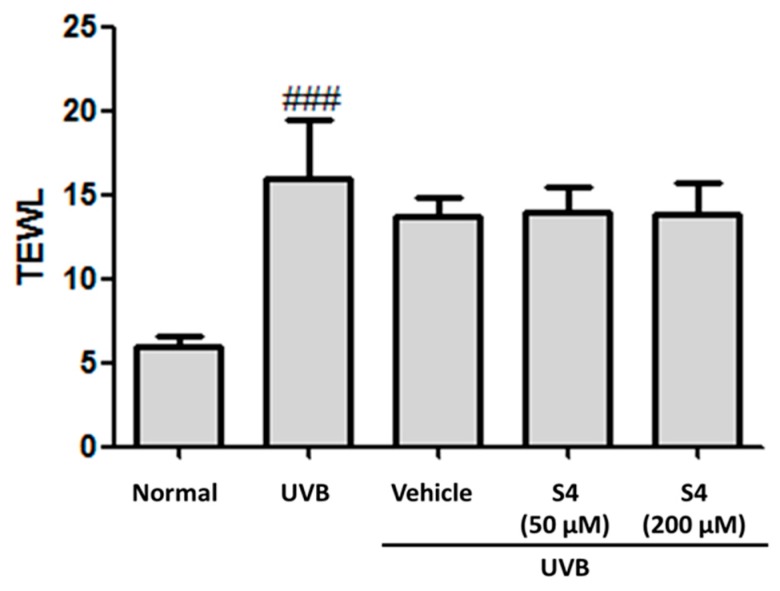
Effect of S4 on transepidermal water loss (TEWL) in chronic UVB-irradiation hairless mice at the 10th week. Significant difference versus normal group: ###, *p* < 0.001.

TEWL is a parameter indicating skin barrier function. TEWL was increased (14.5 ± 0.8 g/h/m^2^) after UVB exposure for 10 weeks (Figure 10). After topical application of S4 to the dorsal skin of hairless mice for 10 weeks, TEWL was 14.3 ± 2.0 g/h/m^2^ (Figure 10). These results signify that S4 was not toxic to the skin.

#### 3.5.3. S4 Reduced UVB-Induced Wrinkle Formation

**Figure 11:**
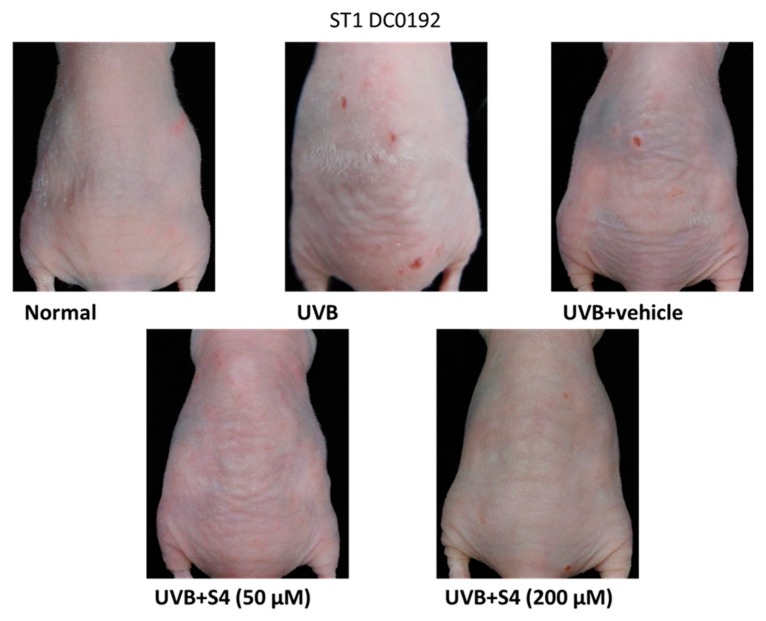
Photographs show skin wrinkles induced by chronic UVB irradiation and the effect of topically applied S4.

Scoring of wrinkle formation on the mouse skin was performed according to the grading scale used in previous research [29,31]. Wrinkle formation was examined macroscopically in the dorsal region and the images were captured by a camera (Figure 11). Topical application of 50 and 200 μM S4 reduced wrinkle formation on the mouse dorsal skin (Figure 11). The wrinkle score was 4.5 ± 1.9 in the UVB-radiated group and substantially decreased to 2.0 ± 1.3 in the 200-μM-S4-treated groups (Table 1). The results indicated that S4 reduced UVB-induced wrinkle formation in the mouse skin.

#### 3.5.4. S4 Ameliorated UVB-Induced Epidermal Hyperplasia

**Figure 12:**
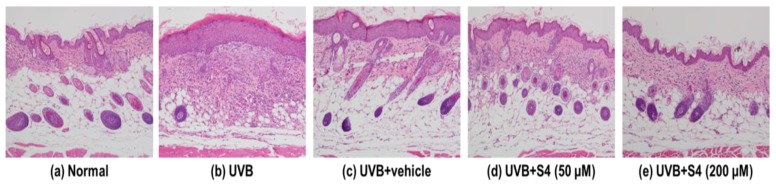
Light micrographs of histological sections stained with hematoxylin and eosin (H&E) of hairless mice.

**Figure 13:**
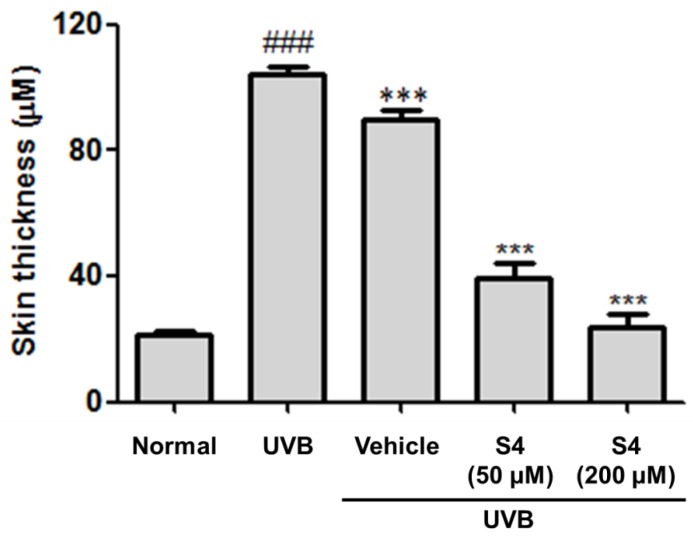
Effect of S4 on skin thickness in chronic UVB-irradiation hairless mice at the 10th week. Significant difference versus normal group: ###, *p* < 0.001. Significant difference versus UVB-irradiation group: ###, *p* < 0.001.

UVB radiation increased the skin thickness of the mice significantly, whereas topical application of S4 reduced the thickness of the skin (Figure 12 and Figure 13). The epidermal thickness was 21.7 ± 1.0 μm in the control group and 104.1 ± 2.4 μm in the UVB-radiated group. The epidermal thickness was 39.2 ± 5.1 μm in the 50-μM-S4-treated group and 24.0 ± 4.1 μm in the 200-μM-S4-treated group (Figure 13). These results suggest that S4 considerably ameliorated UVB-irradiation-induced skin hyperplasia.

#### 3.5.5. S4 Restored Collagen Content in the Dermis

**Figure 14:**
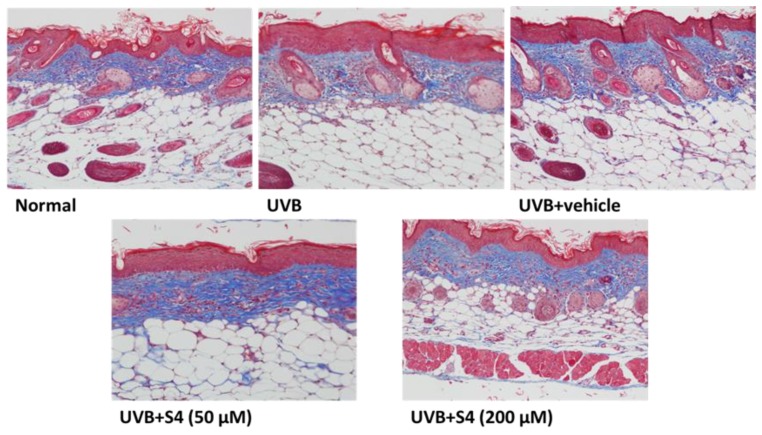
Light micrographs of histological sections stained with Masson’s trichrome in hairless mice. Collagen fibers were stained in blue.

The Masson’s trichrome staining showed that the collagen content in the dermis in the UVB-radiated group was decreased compared with that in the untreated control; however, S4 treatment increased the collagen content in the mouse dermis (Figure 14). These results suggest that S4 restored the collagen content.

#### 3.5.6. S4 Inhibited Photodamage- and Inflammation-Related Protein Levels in UVB-Irradiated Mouse Skin

**Figure 15:**
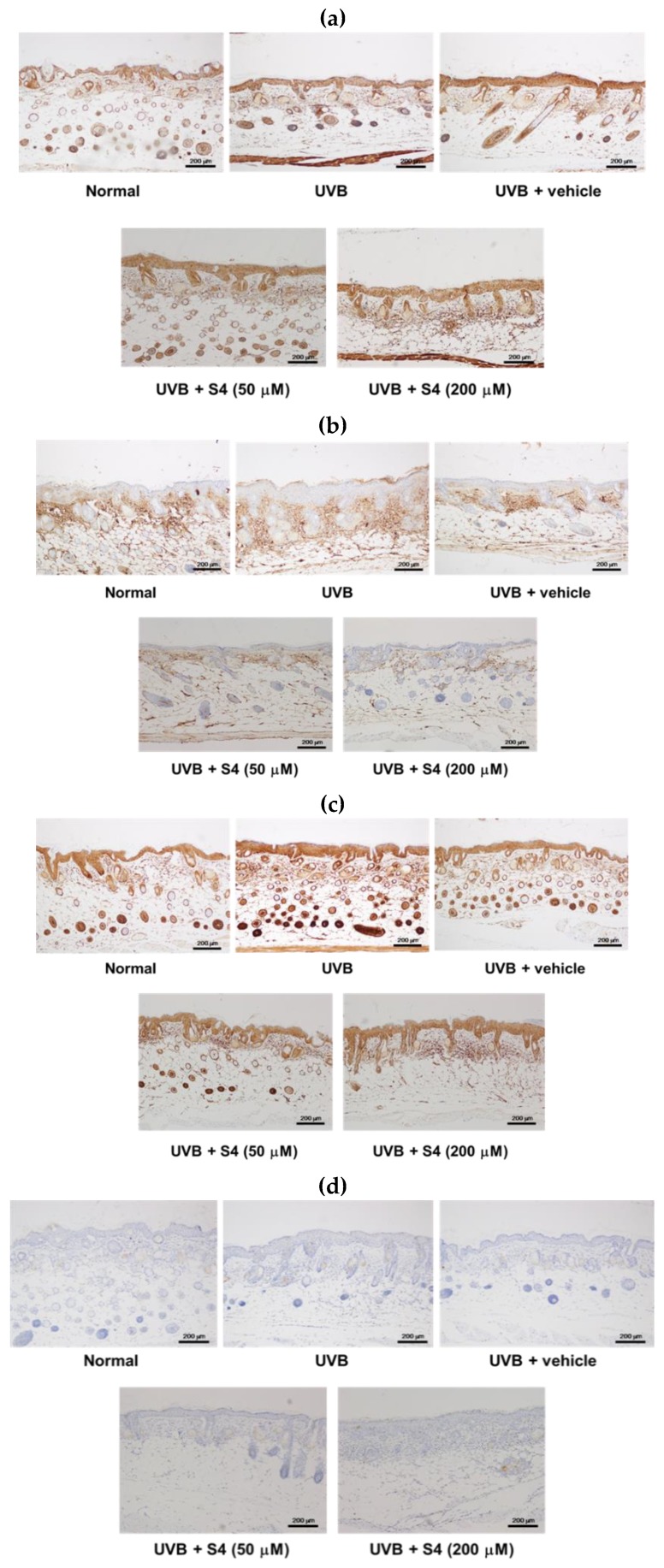
Immunohistological staining of skin slices for (**a**) MMP-1, (**b**) IL-6, (**c**) NF-κB and (**d**) i-NOS in the hairless mouse skin after chronic UVB exposure and S4 treatment.

To understand the molecular factors of S4 on antiphotodamage, photodamage-related proteins were examined in the mouse skin. As shown in Figure 15, MMP-1, IL-6, NF-κB, and i-NOS expression increased in the dermis of hairless mice after exposure to UVB irradiation for 10 weeks, whereas S4 treatment reduced protein expressions (Figure 15). These results indicate that UVB-induced MMP-1 overexpression caused collagen degradation in the skin and that S4 reversed this effect. UVB induced inflammation-related proteins, including IL-6, NF-κB, and i-NOS, whereas S4 treatment inhibited these effects. The results observed in mice further demonstrated those observed in human skin fibroblasts.

## 4. Discussion

UV exposure causes production of ROS and the increase of oxidative stress triggers aging-related signal transduction, resulting in skin sagging, hyperpigmentation, and even skin cancers [30,32]. Natural products and materials that exert antioxidant activity are commonly used in the prevention of photoaging [33]. Sesamin has been reported to exhibit antioxidative and free-radical-scavenging activities, preventing brain and neuron cells injuries [34,35]. Previous research also found that feeding a mixture of sesamin and tocopherol to hairless mice ameliorated UVB-induced sunburn [36]. In one previous study, S4 attenuated ROS generation in hypoxic BV-2 and PC12 cells [23]. In the present study, S4 inhibited UVB-induced ROS formation in skin fibroblasts; the results confirmed the antioxidative activity of S4. 

UV exposure increased ROS generation and NF-ĸB activation, resulting in COX-2 and i-NOS protein expression, subsequently causing skin erythema and inflammation. In previous research, sesamin inhibited tumor necrosis factor-alpha, IL-1, IL-6, NO, COX-2, and i-NOS levels induced by lipopolysaccharide (LPS) in BV-2 microglia [22]. Sesamin also protected neurons from LPS damage by inhibiting the p38 MAP kinase pathway and NF-ĸB activation [21]. UVB can activate the p38 pathway to induce skin inflammation and can even lead to cancer [37,38,39,40]. A previous study reported that sesamin inhibited inflammation of neurons in rats with intracerebral hemorrhage by suppressing ERK and p38 activation [41]. The results of this study indicated that UVB upregulated COX-2 and i-NOS protein expression and NF-ĸB translocation in human skin fibroblasts, whereas S4 inhibited these effects. Additionally, cotreatment with JNK or p38 inhibitor and S4 substantially reduced the protein expression of COX-2; thus, S4 may inhibit COX-2 expression through the JNK and p38 pathways, resulting in anti-inflammation. The results of this study indicated that S4 exhibited anti-inflammatory activity to reduce UVB exposure-induced skin damage.

Erythema (indicated by a* value) increased after UVB exposure, indicating inflammation of the mouse skin [29]. The results of this study indicated that treatment with S4 ameliorated UVB-induced skin inflammation. In addition, the expression of inflammation-related markers, including IL-6, i-NOS, and NF-κB were increased after UVB exposure; however, S4 treatment attenuating the effect. S4 treatment reversed UVB-induced skin erythema and inflammation. Brown granulates were present in the epidermis after UVB exposure, indicating the infiltration of immune cells. S4 reduced the infiltration of leukocytes in the skin. The results of this study indicate that S4 inhibited UVB-induced skin inflammation. In a previous study, S4 inhibited PGE_2_ production induced by hypoxia in BV-2 cells [23]. This study demonstrated the anti-inflammatory effects of S4 by in vitro and in vivo models.

Collagen is secreted by skin fibroblasts and collagen fibers to form collagen fiber bundles to construct the structure of the dermis, conferring resilience and strength to the skin [3]. Long-term exposure to UV causes a decrease of collagen content through increasing the degradation and reducing the synthesis of collagen. Fibroblasts also secrete MMPs, which degrade collagen, and MMP content increases in senescent cells [7]. TIMP is the natural inhibitor of MMPs and the homeostasis between TIMP and MMP plays a criteria role in skin structure and function. The transforming growth factor beta (TGF-β)/Smad pathway regulates the synthesis of collagen; whereas MMPs and the AP-1 pathway play a key role in collagen degradation [42]. The TGF-β pathway is the major pathway regulating collagen synthesis. TGF-β activates Smad2 and Smad3 binds with Smad4 to form a complex which enters the nucleus to subsequently synthesize type I procollagen [43]. UVB radiation reduced Smad3 expression and increased Smad7 expression which may lead to the suppression of collagen biosynthesis. The results of this study found that S4 increased Smad3 expression and inhibited Smad7 overexpression to increase the content of total collagen. S4 restored UVB-induced collagen degradation through ROS scavenging, MMP inhibition, and anti-inflammatory activities. 

UV induces activation of MAP kinase components, including ERK, JNK, and p38, and regulates AP-1, leading to increased MMPs expression and increased collagen digestion [5]. ROS can induce cell damage by upregulating MAP kinase expression. Oxidative stress induced by UV radiation is a pivotal trigger of MMP upregulation by both the AP-1 dependent and independent (p38 activity) pathways [44]. Sesamin also reduced lead and LPS-induced JNK and p38 MAP kinase expression in serum of rats [22]. In previous research, S4 inhibited hypoxia-induced JNK MAP kinase and JNK mRNA expression in PC12 cells [23]. In this study, S4 inhibited UVB-induced AP-1 and the phosphorylation of ERK, JNK, and p38 proteins in human skin fibroblasts. Moreover, UV irradiation inhibited collagen biosynthesis in fibroblasts. The results of this study showed that S4 treatment could block UVB-induced collagen degradation by inhibiting MMP-1, -3, and -9 expression in human skin fibroblasts and decrease MMP-1 content in mouse skin. S4 also elevated TIMP-1 expression against MMP activity. These results demonstrated that S4 exhibited potent antioxidant activity and downregulated MAP kinases and AP-1 expression, ameliorating wrinkle formation induced by chronic UVB exposure. 

In this study, chronic exposure to UVB for 10 weeks caused substantial wrinkle formation on mouse dorsal skin; however, S4 topically applied on the dorsal skin reduced UVB-induced wrinkle formation as long as UVB-induced epidermal thickness decreased significantly by S4 treatment. UVB decreased collagen in the dermis whereas S4 considerably increased the content and density of collagen in the dermis, which may have reduced UV-induced skin damage. 

TEWL is one of the indexes used for measuring skin barrier function and TEWL increases when the stratum corneum is damaged [45]. Oxidative stress, inflammation, and UV exposure have been demonstrated to elevate TEWL. In this study, TEWL was not significantly changed after S4 treatment for 10 weeks, indicating that S4 did not cause toxicity or irritation to the skin. 

## 5. Conclusions

**Figure 16:**
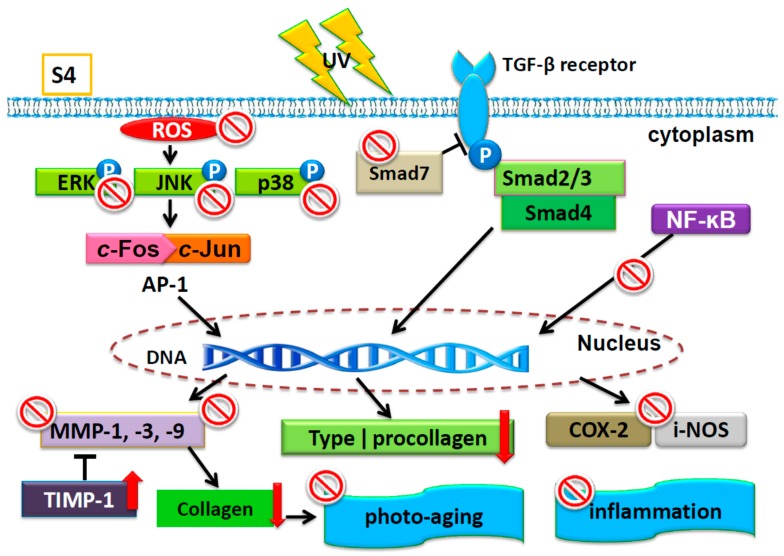
Schematic diagram of S4 ameliorated UVB-induced skin damage.

S4 reduced UVB-induced ROS generation and downregulated MAP kinase and MMP expression, resulting in an increase of collagen content in Hs68 and hairless mice. S4 also inhibited COX-2 and i-NOS protein expression through MAP kinase inhibition to reduce NF-ĸB translocation, resulting in anti-inflammation induced by UVB exposure. S4 decreased UVB-induced wrinkle formation and erythema to protect skin photodamage. The effect and regulation of S4 ameliorated UVB-induced skin damage was shown in Figure 16.

## Figures and Tables

**Table 1:** Effect of sesamin on skin wrinkles induced by UVB irradiation in hairless mice.

	Wrinkle Score (10 Weeks)
Normal mice	1.3 ± 1.2 ^a^
UVB-irradiated mice	4.0 ± 1.6 ^b^
UVB-irradiated mice + vehicle	4.0 ± 2.0 ^bc^
UVB-irradiated mice + S4 (50 μM)	1.3 ± 1.6 ^ad^
UVB-irradiated mice + S4 (200 μM)	2.0 ± 1.3 ^acd^

Values not followed by a common letter are significantly different (*p* < 0.05).

## Abbreviations

AP-1	activator protein-1
COX	cyclooxygenase
DAPI	4′,6-diamidino-2-phenylindole
DCFDA	2′,7′-dichlorofluorescein diacetate
DMEM	Dulbecco’s modified Eagle’s medium
ECM	extracellular matrix
FBS	fetal bovine serum
IL	interleukin
iNOS	inducible nitric oxide synthase
IκB	inhibitor κB
MAP kinase	mitogen-activated protein kinase
MMP	matrix metalloproteinase
MTT	3-[4,5-dimethylthiazole-2-yl]-2,5-diphenyltetrazolium bromide
NF-κB	nuclear factor-kappa B
NO	nitric oxides
ROS	reactive oxygen species
S4	1,2-bis[(3-methoxyphenyl)methyl]ethane-1,2-dicarboxylic acid
SDS	sodium dodecyl sulfate
TEWL	transepidermal water loss
TIMP	tissue inhibitor of metalloproteinase
UV	ultraviolet
